# Milk as a Vehicle for Quinine

**Published:** 1878-10

**Authors:** 


Milk as a Vehicle for Quinine.—New remedies: R. L. Batterbury, M. B., says if one grain of sulphate of quinia be dissolved in an ounce of milk, its bitterness will be hardly perceptible. Even with two grains the bitterness is not marked. Five grains may be taken in two ounces of milk without an unpleasantly bitter taste, while in a tumblerful the bitterness of this quantity is almost lost. Since the doctor chooses to recommend this as a mode of administering quinia to children, we think it proper to say that bad-tasting drugs, like quinia, should be given in articles of food, even in the case of adults, only in exceptional instances. There are other and quite as effectual ways of administering them, and it is therefore hardly advisable to risk the disgust which children often and adults sometimes acquire for wholesome articles of food, in consequence of their having some time been used as vehicles for medicine.—*Louisville Med. News.*

Treatment of the Bromide Eruption.—Dr. Russell reports the case of an epileptic, on whom a generalized, painful pustular eruption broke out, while under treatment, by the bromide of potassium. She was at the time taking about four scruples of the bromide per diem. The eruption was at first papular, but soon became pustular. The pustules were all as large as a pea, and some of them as large as two peas; the base was surrounded by an inflammatory zone. When the bromide was stopped the eruption disappeared, but it reappeared as soon as the medicine was resumed. Dr. Russell at last added five drops of the liquor arsenicalis to each dose of the bromide; in a week the eruption had disappeared, and no new pustules were afterwards developed.—*British Med. Jour.*

---

Lactopeptine.—Our readers may remember that last winter we published the experience of several physicians quite favorable to the above preparation, (Vol. xxxvi, p. 245). Since then we have employed it in several cases of obstinate dyspepsia, and have been gratified, even surprised, at the very excellent results obtained in the great majority of cases. We think it decidedly superior to any form of Pepsin, "pure and simple," we have yet exhibited· *Medical and Surgical Reporter, Philadelphia, Feb. 2d,* 1878.

---

Advantages of Legal Control over Prostitution.—The classical work of Parent Duchatelet, on Prostitution in Paris, contains this passage: "If legislation cannot render men virtuous; if it cannot correct the judgment and repress the impetuosity of passions which appeal to their senses too loudly to leave them the consciousness of duty; at least it may meet the danger to which the imprudent expose themselves, and, for the sake of these men's wives and children, look after the health of the guilty in order to preserve the innocent. I will go further, for I maintain that it ought to do so, and that those who have neglected this important duty have been unfaithful to their trust, and can only be excused by their ignorance of the benefits of the sanitary surveillance of prostitution."—*Medical and Surgical Reporter.*

